# Root exudate-derived compounds stimulate the phosphorus solubilizing ability of bacteria

**DOI:** 10.1038/s41598-023-30915-2

**Published:** 2023-03-10

**Authors:** Hugo A. Pantigoso, Daniel K. Manter, Steven J. Fonte, Jorge M. Vivanco

**Affiliations:** 1 Department of Horticulture and Landscape Architecture, Center for Root and Rhizosphere Biology, Colorado State University, Fort Collins, CO USA; 2 United States Department of Agriculture – Agricultural Research Services, Soil Management and Sugar Beet Research, Fort Collins, CO USA; 3 Department of Soil and Crop Sciences, Colorado State University, Fort Collins, CO USA; 4 Institute of Biology, Leiden University, Sylviusweg 72, 2333 BE Leiden, Netherlands

**Keywords:** Biotechnology, Microbiology, Plant sciences, Environmental sciences

## Abstract

Low phosphorus (P) availability in soils is a major challenge for sustainable food production, as most soil P is often unavailable for plant uptake and effective strategies to access this P are limited. Certain soil occurring bacteria and root exudate-derived compounds that release P are in combination promising tools to develop applications that increase phosphorus use efficiency in crops. Here, we studied the ability of root exudate compounds (galactinol, threonine, and 4-hydroxybutyric acid) induced under low P conditions to stimulate the ability of bacteria to solubilize P. Galactinol, threonine, and 4-hydroxybutyric acid were incubated with the P solubilizing bacterial strains *Enterobacter cloacae*, *Pseudomonas pseudoalcaligenes*, and *Bacillus thuringiensis* under either inorganic (calcium phosphate) or organic (phytin) forms of plant-unavailable P. Overall, we found that the addition of individual root exudate compounds did not support bacterial growth rates. However, root exudates supplemented to the different bacterial appeared to enhance P solubilizing activity and overall P availability. Threonine and 4-hydroxybutyric acid induced P solubilization in all three bacterial strains. Subsequent exogenous application of threonine to soils improved the root growth of corn, enhanced nitrogen and P concentrations in roots and increased available levels of potassium, calcium and magnesium in soils. Thus, it appears that threonine might promote the bacterial solubilization and plant-uptake of a variety of nutrients. Altogether, these findings expand on the function of exuded specialized compounds and propose alternative approaches to unlock existing phosphorus reservoirs of P in crop lands.

## Introduction

Most of the existing phosphorus (P) in soils globally is locked in primary minerals, absorbed on soil particle surfaces, or occurs in organically complexed forms^1,2^. Although P fertilizer is readily available for plants, once applied to soils, it faces constraints such as poor diffusion, limited solubility, and fixation on mineral surfaces; thus, increasing the pool of plant unavailable P in soil^3^.

Phosphate fertilizer originates from rock phosphate minerals, a non-renewable resource that is predicted to become scarce in the coming decades^4,5^. It has been estimated that unlocking residual P pools in soils can play an important role in reducing global P fertilizer demand by up to 50% by 2050^6^. Current strategies to access unavailable soil P and nutrient management practices to supply P to crops are often inefficient. Excessive applications of phosphate fertilizer to agricultural soils are common to overcome soil P fixation processes, and to maintain P in the soil solution at optimal levels^7^. Overapplication of P often leads to increased pollution and decreased farm profitability. Thus, finding widely applicable and sustainable solutions to the inefficiencies in agricultural P use and its bioavailability offers great promise to support long-term productivity and the sustainability of agricultural systems.

The desire to increase P bioavailability in soils has encouraged the study of phytochemicals and beneficial microbes in the plant rhizosphere to enhance P uptake and plant yield^8^. Plant roots can exude a considerable amount of photosynthates within the rhizosphere and this leads to the proliferation of microorganisms within, on the surface, and outside the roots^9,10^. The diverse chemical composition of root exudates contributes to multiple functions including the direct solubilization and acquisition of non-soluble nutrients from the soil and regulation of plant–microbe interactions involved in nutrient acquisition^11^.

Plants possess the ability to modulate the chemical composition of root exudates, that in turn, influence members of the rhizosphere microbial community by discriminating between mutualist, commensal, and pathogenic root-microbe interactions^12,13^. For instance, plants associate with symbiotic and free-living organisms that help mediate plant P uptake; these organisms can be multicellular such as mycorrhizal fungi or single-cell bacteria such as those from the genera *Enterobacter* spp., *Bacillus* spp., or *Pseudomonas* spp.^2^. Plants often initiate these interactions under conditions of soil P limitation^14^, and such interactions are affected by soil type and abiotic factors^15,16^.

The main mechanisms by which plants deal with P scarcity include changes in root morphology by modifying root branching, increasing root length, forming of root hairs, and generally investing more in belowground allocation to increase the root surface for P uptake^17,18^. However, even when plant roots can physically reach the immobile P in soils, this P is often in non-soluble forms that cannot be taken up. The root then switches to complementary strategies to improve solubilization such as the release of selected root exudates to improve P mobilization^19,20^. Some of the major chemical groups of P-mobilizing root exudates include organic acids, such as amino acids and fatty acids, with a range of reported biological functions in the plant rhizosphere^7,13^. P dissolution rates can be greatly accelerated in soil in the presence of organic acids leading to 10–1000-fold higher P concentration in the soil solution, depending on soil type and organic acid concentration^20,21^.

Root exudates can induce the growth of microorganisms, act as chemo-attractants to motile microbes and are a source of carbon (C) for numerous microbes^22,23^. Some bacteria dominate the rhizosphere of certain plants based on specific metabolites secreted by a plant species. For instance, *Burkholderia* species that metabolize citrate and oxalate have been shown to be highly abundant in the rhizosphere of densely packed lateral roots of lupine^24^. The artificial addition of phytochemicals to soils has also been shown to affect the composition and functions of soil microbiota^25–27^. Recent studies have shown that coumarins present in root exudates increase the abundance of single microbial strains or whole microbial communities present in the soil^28,29^. Similarly, the supplementation of soil with organic acids can change the phosphatase enzymatic activity and shift the community composition including beneficial rhizobacteria^30^. In addition, tricarboxylic acids such as malic acid selectively signal and recruit free-living beneficial bacteria *Bacillus subtilis*^31^. Testing the potential enhancement of root exudate-molecules on P solubilizing bacteria (PSB) offers a promising means to increase efficiency of commercial microbial inoculants already in use in farming systems as well as to improve P use efficiency by unlocking legacy P in soils.

In a recent study, Pantigoso et al.^32^ found that certain molecules were exuded in high amounts by *Arabidopsis thaliana* roots grown under deficient P conditions. Some of those molecules containing organic acids directly solubilized non-soluble P under in vitro conditions. In the same study, a second group of molecules such as galactinol, threonine, and 4-hydroxybutyric acid were equally enriched but did not increase P solubilization directly. It was hypothesized that these compounds were involved in signaling with PSB.

The objective of this study was to determine the role of specialized metabolites, previously screened^32^, on the growth and activity of rhizosphere beneficial bacteria. We used corn as a model plant due to its importance as staple food crop. Here we hypothesize that galactinol, threonine, and 4-hydroxybutyric acid, exuded by plants under conditions of P deficiency, can be used to stimulate the growth and/or activity of specific PSB, thus improving the effectiveness of the bacterial inoculum. Further, we tested the possibility that root exudate-derived and specialized metabolites could positively stimulate the native PSBs contained in a natural soil; thus, facilitating nutrient acquisition for the plant.

## Results

### Effects of root exudates on growth rate of phosphorus solubilizing bacteria

**Figure 1:**
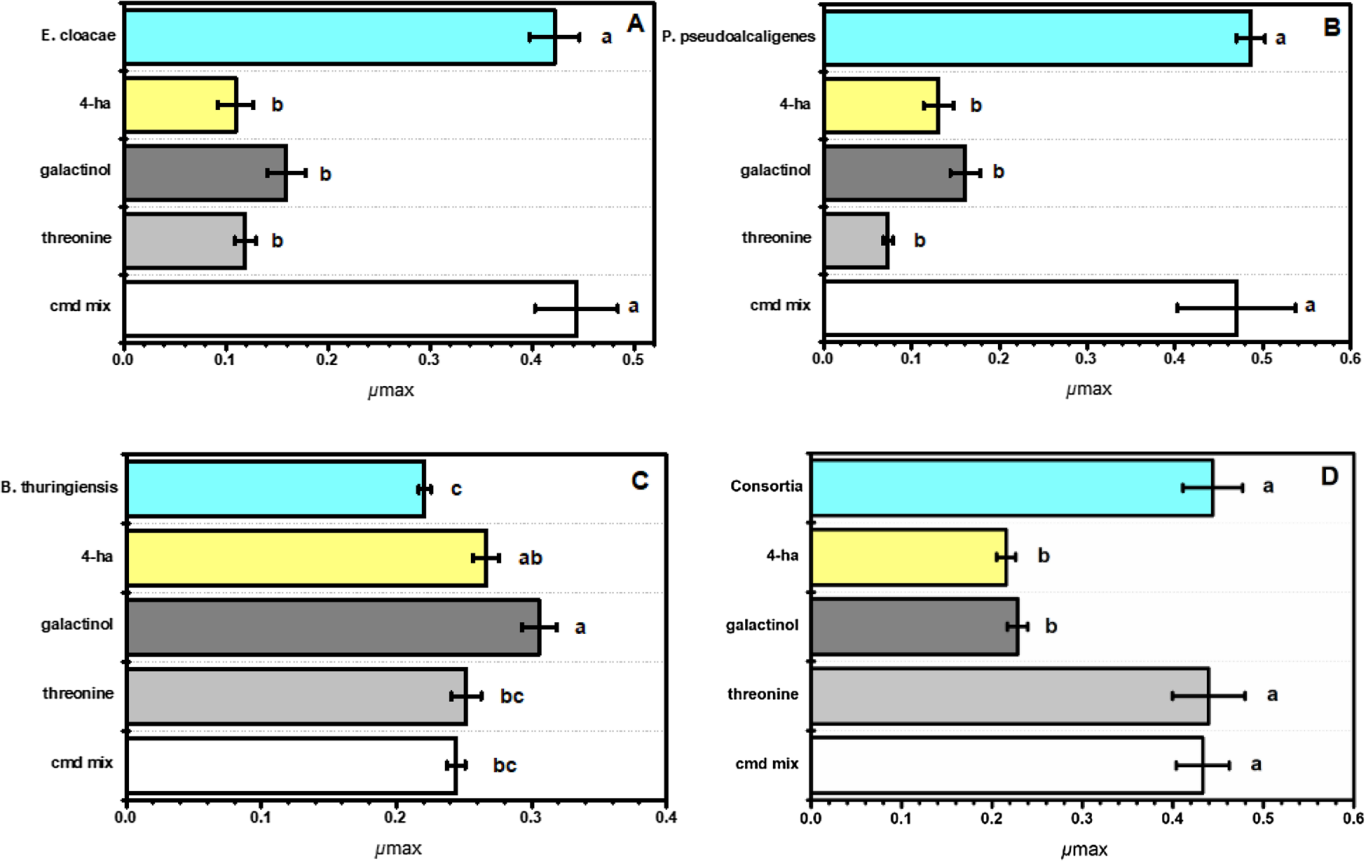
Maximum specific growth rate (*μ*_max_) of P solubilizing bacteria incubated with different root exudates under inorganic calcium phosphate media. Each panel corresponds to (**A**) *Enterobacter cloacae*, (**B**) *Pseudomonas pseudoalcaligenes*, (**C**) *Bacillus thuringiensis*, (**D**) bacteria consortium. Y-axis shows from top to bottom the control: individual bacteria or consortium in blue; 4-ha: 4-hydroxybutyric acid in yellow; galactinol in dark gray; threonine in light grey; and cmd mix: compound combination in white. A one-way ANOVA was performed to compare the effect of root exudates on bacteria. Tukey’s HSD test for multiple comparisons show significant differences. Different letters denote statistical significance among treatments (*p-value* < 0.05).

The effect of the three root exudate-derived compounds was assessed on bacteria growing in an organic and inorganic P media. In the calcium phosphate media, galactinol and 4-hydroxybutyric acid significantly increased the growth rate of *B. thuringiensis,* but threonine, and the combination of compounds did not influence the bacterial growth rate (Fig. 1C). In contrast, the effect of threonine, 4-hydroxybutyric acid, and galactinol significantly decreased the growth rate of *P. pseudoalcaligenes* and *E. cloacae*, but applying a mixture of the compounds did not result in a significant change in growth rate (Fig. 1A,B). Similarly, galactinol and 4-hydroxybutyric acid significantly decreased the growth rate of the bacterial consortia, but no effect was observed for threonine and the combination of the compounds (Fig. 1D).

**Figure 2:**
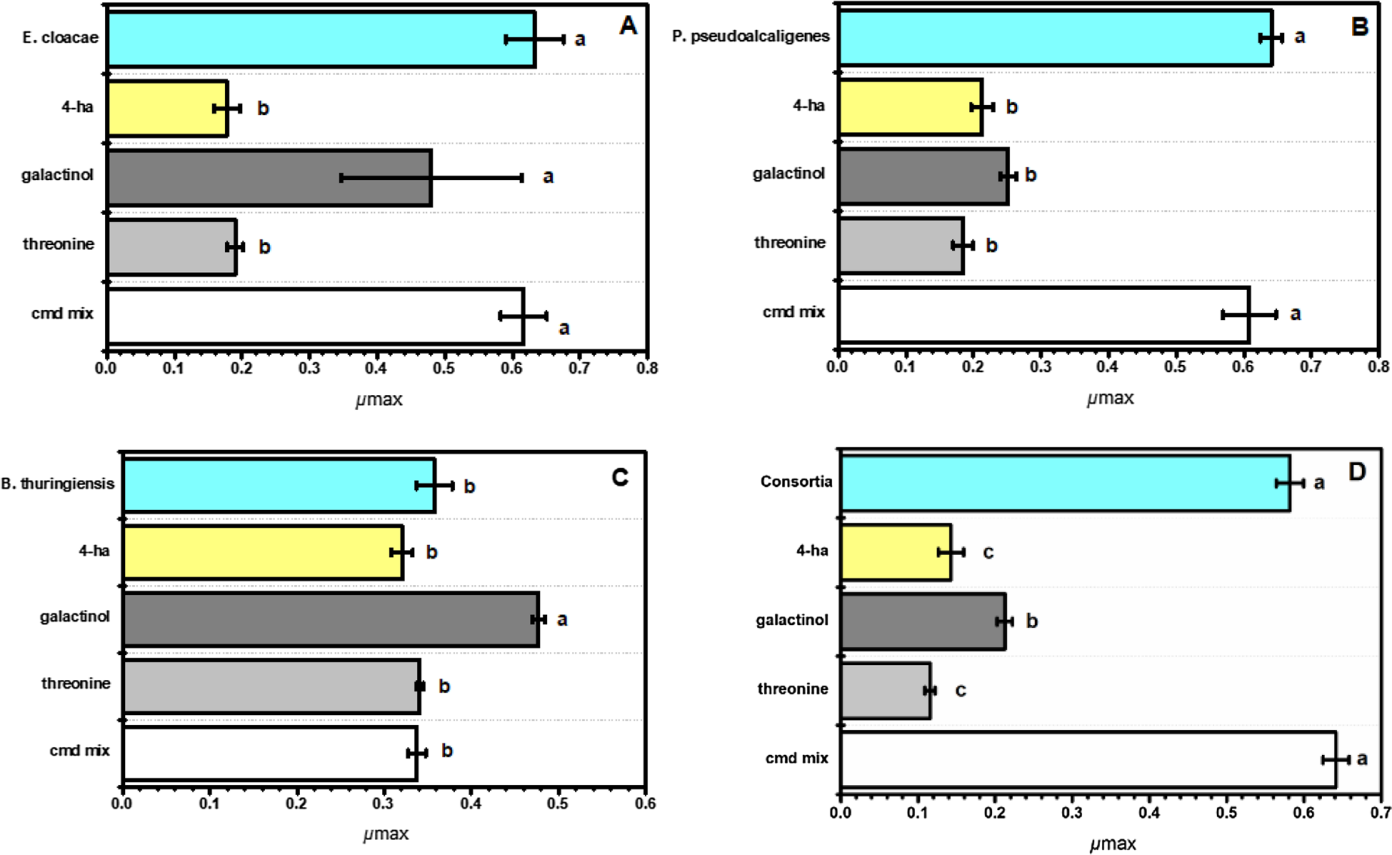
Maximum specific growth rate (*μ*_max_) of P solubilizing bacteria incubated with different root exudates in phytin organic media. Each panel corresponds to (**A**) *Enterobacter cloacae*, (**B**) *Pseudomonas pseudoalcaligenes*, (**C**) *Bacillus thuringiensis*, (**D**) bacterial consortium. Y-axis shows from top to bottom the control: individual bacteria or consortium in blue; 4-ha: 4-hydroxybutyric acid in yellow; galactinol in dark gray; threonine in light grey; and cmd mix: compound combination in white. A one-way ANOVA was performed to compare the effect of root exudates on bacteria. Tukey’s HSD test for multiple comparisons show significant differences. Different letters denote statistical significance among treatments (*p-value* < 0.05).

When examining bacterial growth rate in the organic phytin media, galactinol significantly increased the growth rate of *B. thuringiensis,* but threonine, 4-hydroxybutyric acid and the combination of compounds did not have an effect (Fig. 2C). Similar to what it was observed in the inorganic calcium phosphate media threonine and 4-hydroxybutyric acid significantly decreased the growth rate of *P. pseudoalcaligenes* and *E. cloacae* in the phytin media, but the combination of compounds did not cause a significant change (Fig. 2A,B). Galactinol decreased the growth of *P. pseudoalcaligenes* but did not affect *E. cloacae.* Threonine, 4-hydroxybutyric acid, and galactinol significantly decreased the growth rate of the bacterial consortia but no effect was observed with the combination of compounds (Fig. 2D). In summary, only galactinol showed a significant increase in the growth rate of *B. thuringiensis* under both organic and inorganic P conditions. *E. cloacae* and *P. pseudoalcaligenes* showed significantly reduced growth rate in both P media with all compounds except for the mix, which had a lower concentration of each compound.

### Effects of root exudates on enhancing the phosphorus solubilization ability of bacteria

**Figure 3:**
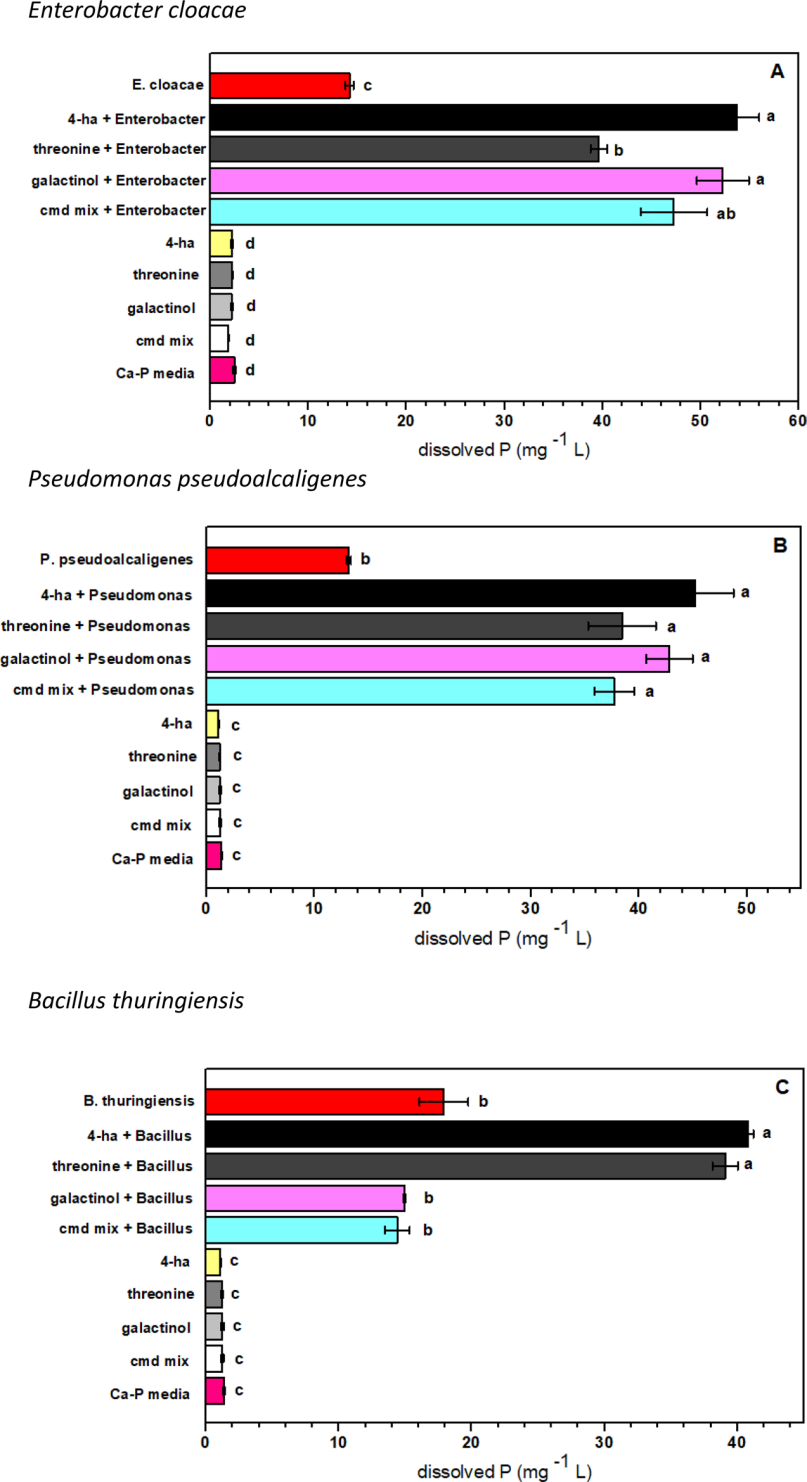

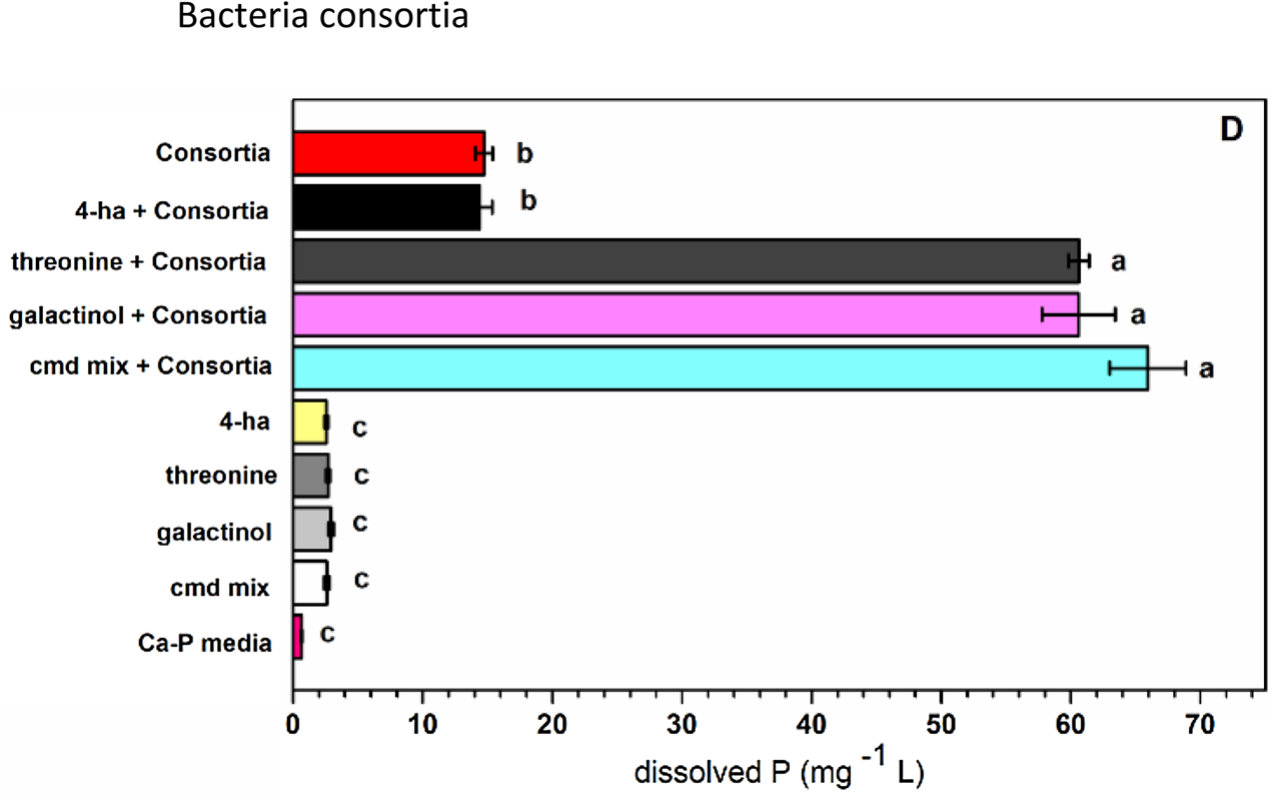
Effect of individual root exudates on dissolved P by phosphorus solubilizing bacteria in a calcium phosphate inorganic media. Each panel corresponds to (**A**) *Enterobacter cloacae*, (**B**) *Pseudomonas pseudoalcaligenes*, (**C**) *Bacillus thuringiensis*, (**D**) Bacteria consortium. A two-ways ANOVA was performed to compare the effect of root exudates on bacteria. Tukey’s HSD test for multiple comparisons show significant differences. Different letters denote statistical significance among treatments (*p-value* < 0.05).

The effect of the three root-exudate derived compounds on the enhancement of P solubilization by bacteria was assessed. In the calcium phosphate inorganic media, threonine, 4-hydroxybutyric acid, galactinol, and the combination of compounds significantly increased dissolved P in the medium for *E. cloacae* and *P. pseudoalcaligenes* (Fig. 3A,B). For *B. thuringiensis*, only threonine and 4-hydroxybutyric acid increased dissolved P (Fig. 3C). In contrast, threonine, galactinol and the combination of compounds significantly increased dissolved P in the bacterial consortia, but 4-hydroxybutyric acid did not (Fig. 3D). In the uninoculated media, there were no significant differences between the added root exudate compounds (Table S1). In phytin (organic phosphate) media, the effect of the compound additions on the enhancement of P solubilization was not significant for any of the bacterial strains (data not shown).

### Effects of root exudate soil amendments on plant biomass

The impact of exogenous application of root-exudate compounds on plant biomass was assessed after periodically adding compounds to corn plants growing in a nutrient-poor soil. Threonine addition significantly increased the fresh root biomass of corn compared to the control treatment (Table 1) but did not influence the shoot or total plant biomass (shoots and roots). The other compounds, galactinol, 4-hydroxybutyric acid, and the combination of compounds, displayed no significant impacts on the corn root, shoot or total fresh biomass (Table 1). We note that while no significant differences were detected (other than for threonine), all treatments receiving the compounds tended to have higher root, shoot and total plant biomass than the control pots (Table 1).

**Table 1:** Fresh weight (g) for shoots, roots and total corn biomass.

Compounds	Shoot biomass	SE (shoot)	Root biomass	SE (root)	Total biomass	SE (total)
Galactinol	3.64	0.238	1.69	0.0836ab	5.33	0.3131
Threonine	3.59	0.233	1.84	0.1343a	5.43	0.3406
4-Hydropropionic acid	3.16	0.208	1.52	0.1031ab	4.68	0.2859
Mix	3.18	0.313	1.59	0.0605ab	4.77	0.3562
Control	3.1	0.230	1.44	0.0636b	4.54	0.2642
p-values	0.369		0.037		0.179	

Table shows mean, and standard error (se) calculated from 10 replicates per treatment. A one-way ANOVA was performed to compare the effect of root exudates on bacteria. Tukey’s HSD test for multiple comparisons show significant differences. Different letters denote statistical significance among treatments (*p-value* < 0.05).

### Effects of root exudates on plant and soil nutrient concentration

**Figure 4:**
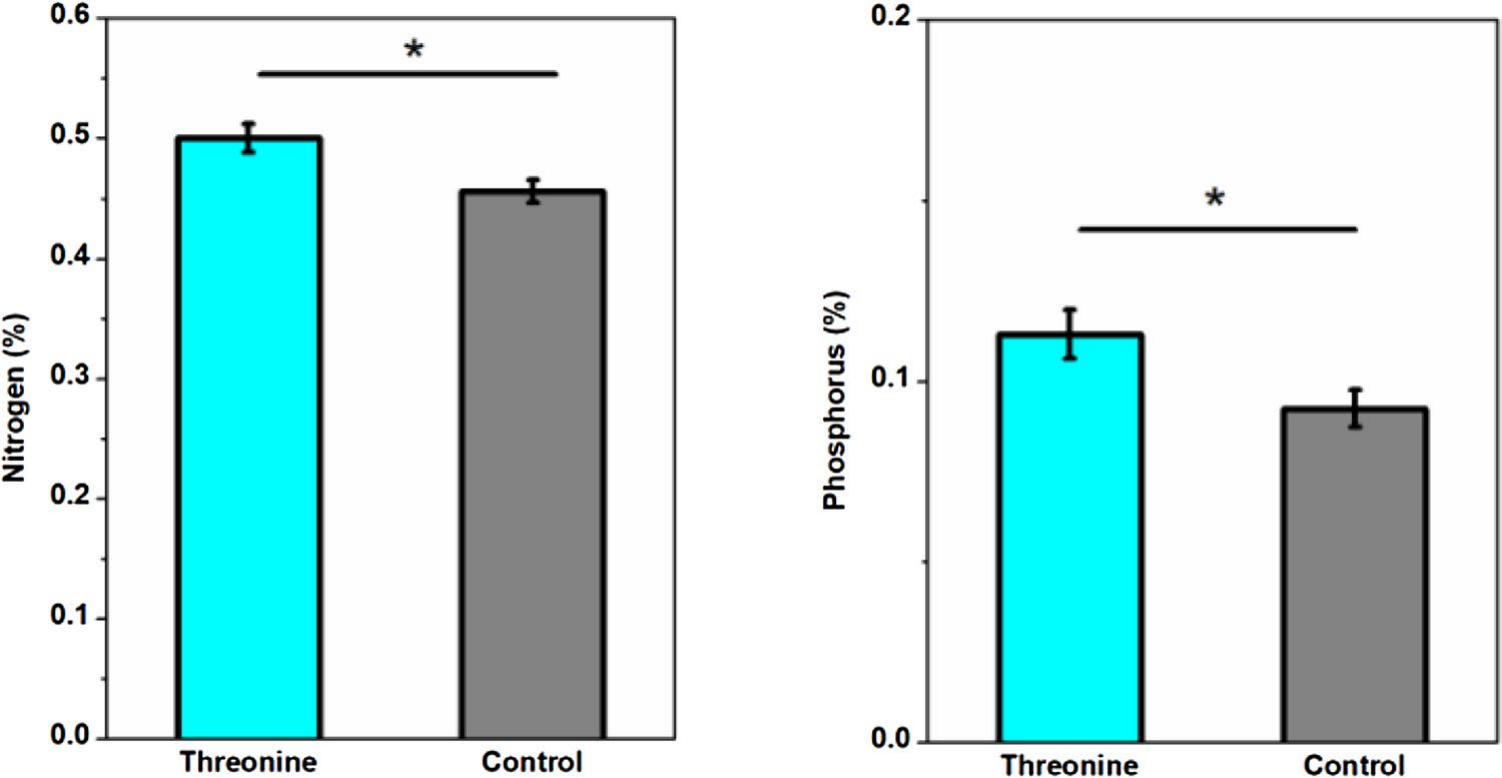
Nitrogen and phosphorus concentrations (%) in corn plant dry roots between exogenous applied threonine and an untreated control. Elemental concentrations were compared by T-test.

Bi-weekly applications of threonine and 4-hydroxypropionic acid increased the concentration of N and P in plant roots related to the untreated control but did not significantly increase the levels of potassium, sulfur, calcium, or magnesium (Fig. 4). Conversely, galactinol and the compound mixture did not affect the concentration of N, P, S, or Ca in root tissues. Galactinol did significantly increase magnesium concentration in roots (Table 2). Effects in nutrient content were also calculated however, not significant differences were found (Table S2).

**Table 2:** Macro and micronutrients concentration in corn roots amended with root exudates.

	Nutrient concentration (%)											
	N	SE (N)	P	SE (P)	K	SE (K)	S	SE (S)	Ca	SE (Ca)	Mg	SE (Mg)
Galactinol	0.643	0.102	0.180	0.050	1.820	0.051	0.203	0.007	0.416	0.013	0.417	0.022
Control	0.456	0.009	0.092	0.005	1.690	0.163	0.172	0.023	0.406	0.026	0.474	0.029
p-value	0.104		0.11		0.257		0.162		0.397		0.074	
Threonine	0.500	0.012	0.113	0.007	1.837	0.091	0.197	0.004	0.430	0.025	0.473	0.027
Control	0.456	0.009	0.092	0.005	1.690	0.163	0.172	0.023	0.406	0.026	0.474	0.029
p-value	0.023		0.037		0.243		0.201		0.286		0.504	
4-Hydroxybutyric acid	0.502	0.002	0.112	0.001	1.923	0.069	0.210	0.003	0.407	0.022	0.441	0.015
Control	0.456	0.009	0.092	0.005	1.690	0.163	0.172	0.023	0.406	0.026	0.474	0.029
p-value	0.016		0.028		0.144		0.122		0.485		0.802	
Mix	0.454	0.006	0.086	0.001	1.760	0.122	0.166	0.010	0.412	0.033	0.502	0.015
Control	0.456	0.009	0.092	0.005	1.690	0.163	0.172	0.023	0.406	0.026	0.474	0.029
p-value	0.556		0.837		0.375		0.591		0.434		0.272	

T-test show comparison of nutrient levels between treatments amended with compounds and an untreated control. Table shows mean, and standard error (se) calculated from 10 replicates per treatment.

The same applications of threonine increased soil available potassium, calcium, and magnesium, but N and P were not significantly altered. The compound 4-hydroxybutyric acid increased calcium and magnesium in soil. Galactinol and the compound combination did not significantly affect K, S, Ca, or Mg levels. Galactinol, 4-hydroxypropionic acid, and the compound combination amended to the soil did not increase N and P content in soils (Table S3).

## Discussion

It has been previously reported that certain root exudates from *A. thaliana* exhibited distinct profiles under different conditions of P availability (sufficient vs. deficient), and that these exudates lead to an increase in dissolved P in a low P environment^23^. In the same study, a second group of compounds were found in high abundance under low P conditions, but no direct enhancement of P-solubilization was observed by those compounds. Thus, we hypothesized that those root exudates must act on P-solubilization via other means. This study investigates whether certain root-derived compounds, under conditions of P scarcity, modulate bacterial functional traits such as growth and P-solubilizing activity. Recent studies have shown that the manipulation of root exudate composition from root apices enriches certain bacterial communities throughout the root system^23^. Here we found that the application of the amino acid threonine, the sugar galactinol, and the fatty acid 4-hydroxybutyric acid, all exudate compounds shown to increase under low P conditions^23^, modulated the growth and activity of PSB strains under in vitro conditions. In addition, our findings suggest that the periodic exogenous amendment of threonine to a natural soil increased the growth of corn roots and increased the levels of plant available K, Mg, and Ca in soils.

We observed bacterial specificity in the effects of the amended compounds. For instance, galactinol increased the growth rate of *B. thuringiensis* but decreased the growth rate of *E. cloacae* and *P. pseudoalcaligenes*. Galactinol and other RFOs (Raffinose Family of Oligosaccharides) are currently emerging as crucial molecules produced by plants during stress responses that provide relief against pathogen infection, drought, and high salinity stress^33,34^. In addition, galactinol has been shown to be used by *Agrobacterium* as a nutrient source providing a competitive advantage to colonize the rhizosphere of tomatoes^35^. The same mechanism to uptake RFOs is highly conserved in bacterial symbionts and pathogens from the Rhizobiaceae family^35^; thus, diverse bacteria appear to have the capability to uptake and metabolize this group of compounds.

It has been reported that high sugar concentrations can inhibit bacterial growth, but lower levels of sugars can exhibit the opposite effect, which indicates that there is a threshold-concentration upon which certain sugars (and other compounds) act as growth inhibitors or as nutrient sources that stimulate growth^36^. When assessing the effect of galactinol on PSB activity we observed that galactinol did not enhance the solubilization of P in *B. thuringiensis* but did increase P solubilization by *E. cloacae*, *P. pseudoalcaligenes*, and in the bacterial consortium. Sugar-like compounds such as galactose and galactosides have been reported to support microbial activity and growth of N-fixing *Sinorhizobium meliloti* before and during nodulation^37^. Zhang et al.^38^ reported that free-living microorganisms in the rhizosphere can use root exudates such as sugars, amino acids, and other compounds to promote colonization and functional traits that support plant growth and nutrition^38^. We note that galactinol increased P-solubilizing activity by *E. cloacae* and *P. pseudoalcaligenes,* but it reduced the growth rate of both bacteria. In contrast, galactinol increased the growth rate of *B. thuringiensis*, while maintaining its P-solubilizing activity. Aforementioned comparisons between bacterial growth rate and P-solubilization were only made under calcium phosphate due to P-solubilization not being significantly affected under the phytin-based media. Galactinol has been shown to be involved as signal molecule that can stimulate root colonization by *Pseudomonas chlororaphis* O6 in cucumber, eliciting an induced systemic resistance against the plant pathogen *Corynespora cassiicola*^34^. When challenged by abiotic stresses such as drought and salinity, tobacco plants overexpressing galactinol synthase (*CsGolS1*) demonstrated improved tolerance, however, bacteria meditation for abiotic stresses was not reported^34^. In light of these findings, we hypothesize that galactinol could be involved in growth rate and P-solubilization activity of PSB and that this effect could be concentration specific. Previous studies have demonstrated that adding C compounds such as glucose to the soil can increase P microbial utilization as compared to solubilization^39,40^, influencing the enrichment of rhizosphere bacteria^41^.

Similar to galactinol, the effect of threonine on PSB growth rate was strain specific. Threonine at 0.1 mM concentration showed an inhibitory effect on the growth rate of *E. cloacae* and *P. pseudoalcaligenes*, but did not affect *B. thuringiensis* in either the organic or inorganic media. Interestingly, treatments with lower amounts of threonine (0.03 mM) from the compound combination did not decrease the growth rate of any of the bacterial strains studied here. Inhibitory effects of amino acids (i.e., cysteine) on *E. coli* at higher concentrations have been previously reported^42^. Despite the negative effect on growth rate, threonine consistently enhanced the P solubilization of all the bacterial strains tested, suggesting a broader effect on PSB strain activity, but not growth rate. In support of this, recent findings show that amino acid metabolism is closely linked to plant–microbe interactions, providing signaling molecules, nutrients, and defense compounds^43^. Amino acids such as threonine are constituents and important N, C or energy sources for growth and activity for a range of bacteria^44^. Further, several bacterial species from the genera *Bacillus*, *Pseudomonas* and *Enterobacter* have been shown to exhibit chemotaxis toward multiple amino acids, including threonine^45,46^. Carvalhais et al.^41^ showed that exudation of different amino acids, in lower amounts, such as asparagine, ornithine, and tryptophan can increase abundance of rhizobacteria *Bacillus* sp. and *Enterobacter* sp. In addition, root exudation of amino acids in P-deficient roots can stimulate the growth and activity of organisms involved in nutrient acquisition^47^. However, the effect of amino acids on bacterial growth and activity are highly variable among bacterial species and is influenced by the environment and the physiology of the organism^48^. Furthermore, bacterial growth inhibition, attraction, and repellent responses are caused by certain amino acids, and these effects are often reversed when the concentration decreases; thus, suggesting the inability of some bacterial strains to metabolize higher concentrations of certain amino acids^48^. For instance, Brisson et al.^49^ showed that shikimic and quinic acids were secreted by roots under phosphate stress and were preferentially absorbed by microorganisms and correlated with root growth^2^. Similarly, Harbort et al.^50^ showed that coumarins improve plant performance by eliciting microbe-assisted iron nutrition. Lin et al.^51^ demonstrated that succinic acid and malonic acid altered the expression of functional genes of Enterobacter sp. PRd5 by increasing the concentration of pyrene degrading enzymes. In addition, organic acids triggered regulation of genes including signal transduction, energy metabolism, and carbohydrate and amino acid metabolisms^51^. These findings suggest that plants can selectively modulate their root exudation profile to stimulate the proliferation of groups of microorganisms that aid in P acquisition.

The effect of 4-hydroxybutyric acid (4-HA) on bacterial growth rate followed the pattern observed for threonine. 4-HA also reduced the growth rate of the bacterial consortia under calcium phosphate media but positively impacted P solubilization in all three PSB strains except for the bacterial consortia. Hydroxy fatty acids such as 4-HA function as modulators of many signal transduction pathways in plants in response to different stresses^52,53^. Recent studies evidenced that fatty acids from plant root exudates have the ability to participate in strong plant–microbe interactions, stimulating N metabolism in rhizosphere bacteria^54^. Lu et al.^55^ demonstrated stimulation of bacterial enzymatic-mediated denitrification by fatty acid oleamide and erucamide from duckweed root exudates.This evidence supports the hypothesis that compounds such as threonine and 4-HA could be acting as a signal rather than simple C source for certain plant beneficial bacteria^43^. We also noted that exogenous application of threonine to soils resulted in an increase of fresh corn root weight, while the other compounds applied did not affect plant growth. We hypothesize that the effect of threonine on plant biomass is a response to its ability to trigger activity and chemotaxis on a wide range of microbes favoring positive nutritional feedback for plants. In support of this hypothesis, a study by Harbort et al.^50^ used plant fitness data, coupled with elemental content and transcriptomic analysis, to confirm that the benefits conferred by commensal microbes under iron limitation occur via a coumarin signaling-molecule mechanism relieving iron starvation. It is commonly held that plants and rhizosphere microbes consume and compete for free amino acids in the rhizosphere^56,57^. Plant roots are often outcompeted by microbes in the uptake of externally applied amino acids^58,59^. These observations have led to the speculation that amino acids may be taken up from the rhizosphere, where they are first rescued and mineralized by bacteria, and then used as an inorganic N source by plants^59^. In addition, under nutrient limited conditions bacterial survival strategies can increase their ability to catabolize amino acids^60^. We found that threonine increased N and P concentration in plant root tissues, and the available Ca and Mg in soils were higher as well.

It was also found that bacterial growth response was similar under organic and inorganic P, but the P-solubilizing activity varied. The three compounds tested impacted PSB activity under calcium phosphate but did not affect P solubilization under phytin. It has been reported that the ability of microbes to solubilize P is highly dependent on the source of P^61,62^. Thus, it appears that threonine, galactinol and 4-hydroxybutyric acid are inducing mineral dissolving compounds such as organic acids that help the bacteria to solubilize inorganic P. This is in contrast to the mechanism used by bacteria to solubilize/mineralize organic P such as the secretion of phosphatases and phytases^63^. Lastly, this research expands on the potential application of specialized root exudate compounds that could lead to agricultural technologies such as its use as elicitors of indigenous bacteria fostering beneficial association with plant roots that positively impact health and productivity.

## Conclusion

Specialized metabolites, derived from root exudates, act as signals and sources for rhizosphere microorganisms with implications for P availability and uptake by plants. This study has examined the effects of specialized root exudates, such as threonine, 4-hydroxybutyric acid and galactinol and their ability to stimulate P-solubilizing activity of bacteria as well as implications for soil and plant nutrient uptake. Effects of specialized compounds on bacteria were found to be species and P source dependent. Under greenhouse conditions, threonine was shown to stimulate root growth and, together with 4-hydroxybutyric acid, result in significantly higher N and P content in root tissues. Our findings expand on the function of exuded specialized compounds and suggest alternative approaches to effectively recover residual P from soil. Further work should focus on identifying and testing root exudate-derived compounds aiming to efficiently promote biological activity, growth and functional features, leading to improvements in nutrient use efficiency, and the reduction of excessive applications of synthetic fertilization to croplands.

## Methods

### Phosphorus solubilizing bacteria and root-exudate derived compounds

This study used bacterial strains *Enterobacter cloacae*, *Bacillus thuringiensis*, and *Pseudomonas pseudoalcaligenes* that were isolated from wild potato (*Solanum bulbocastanum*) and previously screened for their ability to solubilize P and tested in vitro and *in planta* experiments^14,64^. Similarly, this study employed three root exudate-derived compounds: galactinol, threonine, and 4-hydroxybutyric acid, that were identified previously to occur in high concentrations in the root exudation profile of *Arabidopsis thaliana* grown under low P conditions^32^.

### Effect of root exudates on bacterial growth

The objective of this experiment was to measure the effects of root exudate-derived compounds on PSB growth with different sources of unavailable P. Five bacteria treatments (*E. cloacae*, *B. thuringiensis*, *P. pseudoalcaligenes,* a consortium of the three strains and a sterile control) and five root exudate treatments (galactinol, threonine, and 4-hydroxybutyric acid, a combination of the three, and a control) were grown in two different P media with low P availability (calcium phosphate or phytin based). In total, there were 50 treatments with 4 replicates per treatment.

A 10 μL diluted (OD_600_ = 1; 1 × 10^8^) aliquot from each pure culture of *E. cloacae*, *B. thuringiensis*, and/or *P. pseudoalcaligenes* and 5 μL of each of the three compounds at 10 mM concentration were combined with 150 μL calcium phosphate or phytin liquid medium separately (one bacterial strain per compound) and in combination (one strain combined with the compound mixture) in a 96-well plate. Subsequently, the plate was incubated for 48 h at 25 °C in a spectrophotometer, and growth, was monitored by optical density (660 nm). After incubation, the maximum specific growth rate for the culture (*μ*_max_) was used to compare the effect of each compound on bacterial growth, based on the calculations of Maier and Pepper^65^. Liquid calcium phosphate/phytin medium without the addition of bacteria was used as a control. Deionized and DNA-free water was used to bring the controls to the same volume as the inoculated treatments.

### Root exudate and bacteria effects on P solubilization

Using the same 50 treatments described above, we tested the effect of the root exudate compounds together with PSB on P solubilization. Using a 2.5 mm platinum wire loop, a streak of bacteria culture obtained from pure cultures of each of the three selected isolates was dipped into liquid Luria–Bertani medium^66^, and incubated separately in a rotary shaker at 170 rev min^−1^ at room temperature overnight until reaching the mid-exponential growth phase. A 50 μL diluted (OD_600_ = 1; 1 × 10^8^) aliquot from each pure bacterial culture grown in an Erlenmeyer flask and 50 μL of 10 mM concentration from a given compound, stored in 15 mL cylindrical tubes, (galactinol, threonine, and 4-hydroxybutyric acid) was added to 4.95 mL liquid NBRIP (National Botanical Research Institute Phosphate) medium, with a final concentration of 0.1 mM, and incubated in a rotary shaker for 72 h^67^. One of each of the three dissolved compounds was combined (one-third part per each compound) and mixed at the same final concentration of 0.1 mM. For the inoculation of the bacterial co-inoculum, one of each of the three bacterial strains was prepared and mixed at the same final concentration (OD_600_ = 1; 1 × 10^8^) and incubated for 72 h. Two plant-unavailable sources of P, calcium phosphate and phytin, were used to prepare NBRIP medium. The NBRIP medium is comprised of glucose (10.0 g), Ca_3_(PO_4_)_2_ (5.0 g), NaCl (0.2 g), MgSO_4_·7H_2_O (0.5 g), (NH_4_)_2_SO_4_ (0.5 g), KCl (0.2 g), MnSO_4_ (0.03 g), FeSO_4_·7H_2_O (0.003 g) with a pH of 7.0–8.0. For phytin media preparation, calcium phosphate was replaced with 10 g of phytin (C_6_H_6_Ca_6_O_24_P_6_). The pH of the initial P media was near neutral for both P media (~ 7 pH). Each bacterium treatment was run in an independent batch, thus non-bacterial control treatment were included with each batch run.

After incubation, the solution was centrifuged at 6000 rpm for 20 min to remove both the suspended bacteria cells and the remaining calcium phosphate/phytate. Sterile, liquid calcium phosphate/phytin medium, with each compound separately, and without the addition of bacteria, were used as controls. The concentration of phosphate in the supernatant was analyzed according to the protocol of Soltanpour et al.^68^ and measured with an inductively coupled plasma-optical emission spectrometer (ICP-OES; Perkin Elmer 7300DV) at the Soil, Water and Plant Testing Laboratory of Colorado State University.

### Impacts of root exudates on soil nutrient availability and plant growth

Certified organic seeds of commercial corn (*Zea mays*) cultivar ‘Natural Sweet F1’ from Johnny’s Selected Seeds (Windslow, Maine) were grown under greenhouse conditions at the Horticulture Center of Colorado State University, Fort Collins, CO. The average temperature in the greenhouse was 20 to 25 °C and the experiment lasted six weeks. Seeds were sown in squared pots (5 cm × 4 cm × 4 cm) containing 300 g pine forest soil, collected to a depth of 30 cm from a natural area (O horizon), Grey Rock Forest, Poudre Canyon, Bellvue, CO, (40.69°N, 105.28°W, 1700 masl). The climate is semiarid, with an average annual precipitation of 409 mm (usclimatedata.com, accessed 2021). The soil is classified as a sandy clay loam with an organic matter content of 3.3%, nitrogen (N) content of 0.4 ppm, available P 26.7 ppm based on AB-DTPA extract, and a pH of 6.8. Pine soil forest with no history of fertilizer amendment was used because of its undisturbed conditions relative to highly managed agricultural soils. No fertilization or amendments were applied, and the corn plants were irrigated based on growth and demand keeping a relatively constant moisture in the soil. Pots with corn plants were assigned to each of five treatments, with 10 repetitions per each treatment. The treatments consisted of pots receiving one individual compound and the three in combination, as well as the control. The compounds galactinol, threonine, 4-hydroxybutyric acid, and a combination were applied to the base of the corn plants twice a week. A volume of 1 mL at 1 mM concentration was added to pots each time, except for the control, which received an equivalent amount of pure water. The treatment with the combination of compounds also received addition with a total concentration of 1 mM (0.33 mL of each compound).

Plants were harvested 6 weeks after emergence, roots were gently rinsed to removed soil particles, and the fresh weight of roots and shoots was recorded. Plants were oven dried at 90 °C for 72 h, and the dry weight was also recorded. Total P in the plant shoot and root tissues were analyzed separately by digesting the plant tissue in a block digester with HCl and HNO_3_ and cleared with H_2_O_2_. Then the sample was brought to a volume of 50 mL, and total P was read on an ICP-OES. Available P in the soil samples was identified using the Olsen P method^69^. Both plant and soil N, P, potassium (K), calcium (Ca), and magnesium (Mg) analysis were performed at the Ward Laboratories (Kearney, Nebraska).

### Data analysis

The effect of different root-exudate compounds with and without bacterial strains were compared separately for each bacterium and P media treatment combination using one-way ANOVA. The effects of root-exudate derived compounds on bacterial growth rate were also compared separately for each bacteria treatment with one-way ANOVA. One-way ANOVA was also used to examine the effects of compound addition on plant dry biomass, and P content and other nutrients in soil and plant tissue. Homogeneity of variance and normality were assessed for all analyses. A probability level of *p* = 0.05 was considered statistically significant. A t-test was used to compared nutrient concentration between control and individual compounds.

### Research involving plants statement

Plants used in this study come from organic and certified seeds commercially available. No special permits are required to obtain this seeds.

## Supplementary Information

Supplementary Tables.

## Data Availability

All the data generated or analyzed during this research are included in this published article and its supplementary information files. The datasets used and/or analyzed during the current study available from the corresponding author on reasonable request.

## References

[1] Syers JK, Johnston AE, Curtin D (2008). Efficiency of soil and fertilizer phosphorus use. FAO Fertil. Plant Nutr. Bull..

[2] Richardson AE, Simpson RJ (2011). Soil microorganisms mediating phosphorus availability update on microbial phosphorus. Plant Physiol..

[3] Stutter MI, Chardon WJ, Kronvang B (2012). Riparian buffer strips as a multifunctional management tool in agricultural landscapes: Introduction. J. Environ. Qual..

[4] Cordell D, White S (2015). Tracking phosphorus security: Indicators of phosphorus vulnerability in the global food system. Food Secur..

[5] Cordell D, Drangert JO, White S (2009). The story of phosphorus: Global food security and food for thought. Glob. Environ. Change.

[6] Sattari SZ, Bouwman AF, Giller KE, van Ittersum MK (2012). Residual soil phosphorus as the missing piece in the global phosphorus crisis puzzle. Proc. Natl. Acad. Sci. USA.

[7] Menezes-Blackburn D, Giles C, Darch T, George TS, Blackwell M, Stutter M (2018). Opportunities for mobilizing recalcitrant phosphorus from agricultural soils: A review. Plant Soil.

[8] Richardson AE, Barea JM, McNeill AM, Prigent-Combaret C (2009). Acquisition of phosphorus and nitrogen in the rhizosphere and plant growth promotion by microorganisms. Plant Soil.

[9] Dakora FD, Phillips DA (2002). Root exudates as mediators of mineral acquisition in low-nutrient environments. Food Security in Nutrient-Stressed Environments: Exploiting Plants’ Genetic Capabilities.

[10] Badri DV, Vivanco JM (2009). Regulation and function of root exudates. Plant Cell Environ..

[11] Pantigoso HA, Newberger D, Vivanco JM (2022). The rhizosphere microbiome: Plant-microbial interactions for resource acquisition. J. Appl. Microbiol..

[12] Chaparro JM, Sheflin AM, Manter DK, Vivanco JM (2012). Manipulating the soil microbiome to increase soil health and plant fertility. Biol. Fertil. Soils.

[13] Zhalnina K, Louie KB, Hao Z, Mansoori N, da Rocha UN, Shi S (2018). Dynamic root exudate chemistry and microbial substrate preferences drive patterns in rhizosphere microbial community assembly. Nat. Microbiol..

[14] Pantigoso HA, He Y, Manter DK (2022). Phosphorus-solubilizing bacteria isolated from the rhizosphere of wild potato *Solanum bulbocastanum* enhance growth of modern potato varieties. Bull. Natl. Res. Cent..

[15] Venturi V, Keel C (2016). Signaling in the rhizosphere. Trends Plant Sci..

[16] Sasse J, Martinoia E, Northen T (2018). Feed your friends: Do plant exudates shape the root microbiome?. Trends Plant Sci..

[17] Hermans C, Hammond JP, White PJ, Verbruggen N (2006). How do plants respond to nutrient shortage by biomass allocation?. Trends Plant Sci..

[18] Lynch JP, Brown KM (2008). Root strategies for phosphorus acquisition. The Ecophysiology of Plant-Phosphorus Interactions.

[19] Jones DL, Darrah PR (1995). Influx and efflux of organic acids across the soil-root interface of *Zea mays* L. and its implications in rhizosphere C flow. Plant Soil.

[20] Gerke J (2015). The acquisition of phosphate by higher plants: Effect of carboxylate release by the roots. A critical review. J. Plant Nutr. Soil Sci..

[21] Jones DL, Darrah PR (1994). Role of root derived organic acids in the mobilization of nutrients from the rhizosphere. Plant Soil.

[22] Walker TS, Bais HP, Grotewold E, Vivanco JM (2003). Root exudation and rhizosphere biology. Plant Physiol..

[23] Kawasaki A, Dennis PG, Forstner C, Raghavendra AK, Mathesius U, Richardson AE (2021). Manipulating exudate composition from root apices shapes the microbiome throughout the root system. Plant Physiol..

[24] Weisskopf L, Heller S, Eberl L (2011). Burkholderia species are major inhabitants of white lupin cluster roots. Appl. Environ. Microbiol..

[25] Badri DV, Weir TL, Van der Lelie D, Vivanco JM (2009). Rhizosphere chemical dialogues: plant–microbe interactions. Curr. Opin. Biotechnol..

[26] Badri DV, Chaparro JM, Zhang R, Shen Q, Vivanco JM (2013). Application of natural blends of phytochemicals derived from the root exudates of Arabidopsis to the soil reveal that phenolic-related compounds predominantly modulate the soil microbiome. J. Biol. Chem..

[27] Huang AC, Jiang T, Liu YX, Bai YC, Reed J, Qu B (2019). A specialized metabolic network selectively modulates Arabidopsis root microbiota. Science.

[28] Voges MJ, Bai Y, Schulze-Lefert P, Sattely ES (2019). Plant-derived coumarins shape the composition of an Arabidopsis synthetic root microbiome. Proc. Natl. Acad. Sci. USA.

[29] Koprivova A, Schuck S, Jacoby RP, Klinkhammer I, Welter B, Leson L (2019). Root-specific camalexin biosynthesis controls the plant growth-promoting effects of multiple bacterial strains. Proc. Natl. Acad. Sci. USA.

[30] Macias-Benitez S, Garcia-Martinez AM, Caballero Jimenez P, Gonzalez JM, Tejada Moral M, Parrado Rubio J (2020). Rhizospheric organic acids as biostimulants: Monitoring feedbacks on soil microorganisms and biochemical properties. Front. Plant Sci..

[31] Rudrappa T, Czymmek KJ, Paré PW, Bais HP (2008). Root-secreted malic acid recruits beneficial soil bacteria. Plant Physiol..

[32] Pantigoso HA, Yuan J, He Y, Guo Q, Vollmer C, Vivanco JM (2020). Role of root exudates on assimilation of phosphorus in young and old *Arabidopsis thaliana* plants. PLoS ONE.

[33] Sengupta S, Mukherjee S, Basak P, Majumder AL (2015). Significance of galactinol and raffinose family oligosaccharide synthesis in plants. Front. Plant Sci..

[34] Kim MS, Cho SM, Kang EY, Im YJ, Hwangbo H, Kim YC (2008). Galactinol is a signaling component of the induced systemic resistance caused by *Pseudomonas chlororaphis* O6 root colonization. Mol.Plant-Microbe Interact..

[35] Meyer T, Vigouroux A, Aumont-Nicaise M, Comte G, Vial L, Lavire C, Moréra S (2018). The plant defense signal galactinol is specifically used as a nutrient by the bacterial pathogen Agrobacterium fabrum. J. Biol. Chem..

[36] Mizzi L, Maniscalco D, Gaspari S, Chatzitzika C, Gatt R, Valdramidis VP (2020). Assessing the individual microbial inhibitory capacity of different sugars against pathogens commonly found in food systems. Lett. Appl. Microbiol..

[37] Bringhurst RM, Cardon ZG, Gage DJ (2001). Galactosides in the rhizosphere: utilization by Sinorhizobium meliloti and development of a biosensor. Proc. Natl. Acad. Sci. USA.

[38] Zhang N, Wang D, Liu Y, Li S, Shen Q, Zhang R (2014). Effects of different plant root exudates and their organic acid components on chemotaxis, biofilm formation and colonization by beneficial rhizosphere-associated bacterial strains. Plant Soil.

[39] Kouno K, Wu J, Brookes PC (2002). Turnover of biomass C and P in soil following incorporation of glucose or ryegrass. Soil Biol. Biochem..

[40] Zhang L, Ding X, Peng Y, George TS, Feng G (2018). Closing the loop on phosphorus loss from intensive agricultural soil: A microbial immobilization solution?. Front. Microbiol..

[41] Carvalhais LC, Dennis PG, Badri DV, Kidd BN, Vivanco JM, Schenk PM (2015). Linking jasmonic acid signaling, root exudates, and rhizosphere microbiomes. Mol. Plant Microbe Interact..

[42] Harris CL (1981). Cysteine and growth inhibition of *Escherichia coli:* Threonine deaminase as the target enzyme. J. Bacteriol..

[43] Moormann J, Heinemann B, Hildebrandt TM (2022). News about amino acid metabolism in plant–microbe interactions. Trends Biochem. Sci..

[44] Radkov AD, McNeill K, Uda K, Moe LA (2016). d-Amino acid catabolism is common among soil-dwelling bacteria. Microbes Environ..

[45] Ordal GW, Gibson KJ (1977). Chemotaxis toward amino acids by *Bacillus subtilis*. J. Bacteriol..

[46] Oku S, Komatsu A, Tajima T, Nakashimada Y, Kato J (2012). Identification of chemotaxis sensory proteins for amino acids in Pseudomonas fluorescens Pf0–1 and their involvement in chemotaxis to tomato root exudate and root colonization. Microbes Environ..

[47] Carvalhais LC, Dennis PG, Fedoseyenko D, Hajirezaei MR, Borriss R, von Wirén N (2011). Root exudation of sugars, amino acids, and organic acids by maize as affected by nitrogen, phosphorus, potassium, and iron deficiency. J. Plant Nutr. Soil Sci..

[48] Yang YM, Pollard A, Höfler C, Poschet G, Wirtz M, Hell R, Sourjik V (2015). Relation between chemotaxis and consumption of amino acids in bacteria. Mol. Microbiol..

[49] Brisson V, Richardy J, Kosina S, Northen T, Vogel J, Gaudin A (2021). Phosphate availability modulates root exudate composition and rhizosphere microbial community in a teosinte and a modern maize cultivar. Phytobiomes J..

[50] Harbort CJ, Hashimoto M, Inoue H, Niu Y, Guan R, Rombolà AD (2020). Root-secreted coumarins and the microbiota interact to improve iron nutrition in Arabidopsis. Cell Host Microbe.

[51] Lin C, Zhang F, Sun L, Zhou Z, Chen R, Zhu X (2022). Contribution of dicarboxylic acids to pyrene biodegradation and transcriptomic responses of Enterobacter sp. PRd5. Appl. Microbiol. Biotechnol..

[52] Macabuhay A, Arsova B, Walker R, Johnson A, Watt M, Roessner U (2021). Modulators or facilitators? Roles of lipids in plant root–microbe interactions. Trends in Plant Science.

[53] Siebers M, Brands M, Wewer V, Duan Y, Hölzl G, Dörmann P (2016). Lipids in plant–microbe interactions. Biochim. Biophys. Acta.

[54] Sun L, Lu Y, Kronzucker HJ, Shi W (2016). Quantification and enzyme targets of fatty acid amides from duckweed root exudates involved in the stimulation of denitrification. J. Plant Physiol..

[55] Lu Y, Zhou Y, Nakai S, Hosomi M, Zhang H, Kronzucker HJ, Shi W (2014). Stimulation of nitrogen removal in the rhizosphere of aquatic duckweed by root exudate components. Planta.

[56] Owen AG, Jones DL (2001). Competition for amino acids between wheat roots and rhizosphere microorganisms and the role of amino acids in plant N acquisition. Soil Biol. Biochem..

[57] Forsum O, Svennerstam H, Ganeteg U, Näsholm T (2008). Capacities and constraints of amino acid utilization in Arabidopsis. New Phytol..

[58] Reeve JR, Smith JL, Carpenter-Boggs L, Reganold JP (2009). Glycine, nitrate, and ammonium uptake by classic and modern wheat varieties in a short-term microcosm study. Biol. Fertil. Soils.

[59] Moe LA (2013). Amino acids in the rhizosphere: from plants to microbes. Am. J. Bot..

[60] Zinser ER, Kolter R (1999). Mutations enhancing amino acid catabolism confer a growth advantage in stationary phase. J. Bacteriol..

[61] Illmer P, Schinner F (1995). Solubilization of inorganic calcium phosphates—solubilization mechanisms. Soil Biol. Biochem..

[62] Wan W, Qin Y, Wu H, Zuo W, He H, Tan J (2020). Isolation and characterization of phosphorus solubilizing bacteria with multiple phosphorus sources utilizing capability and their potential for lead immobilization in soil. Front. Microbiol..

[63] Sinha RM (1967). Studies on the effect of Mg2+ ions, citrate and phenylalanine on alkaline phosphatase. Proc. Assoc. Clin. Biochem..

[64] Pantigoso HA, Manter DK, Vivanco JM (2020). Differential effects of phosphorus fertilization on plant uptake and rhizosphere microbiome of cultivated and non-cultivated potatoes. Microb. Ecol..

[65] Maier RM, Pepper IL (2015). Bacterial growth. Environmental Microbiology.

[66] Bertani G (1951). Studies on lysogenesis I: The mode of phage liberation by lysogenic Escherichia coli. J. Bacteriol..

[67] Nautiyal CS (1999). An efficient microbiological growth medium for screening phosphate solubilizing microorganisms. FEMS Microbiol. Lett..

[68] Soltanpour PN, Jones JB, Workman SM (1983). Optical emission spectrometry. Methods Soil Anal..

[69] Olsen SR (1954). Estimation of Available Phosphorus in Soils by Extraction with Sodium Bicarbonate (No. 939).

