# Supplementary material for: Root exudate-derived compounds stimulate the phosphorus solubilizing ability of bacteria
Source: Sci Rep. 2023 Mar 10;13:4050. doi: 10.1038/s41598-023-30915-2 (PMC10006420; doi:10.1038/s41598-023-30915-2)
Supplement: Supplementary file 1 — Supplementary Tables. [file 41598_2023_30915_MOESM1_ESM.docx]

**Supplementary Material Table 1.** Effects of root exudates on phosphorus solubilizing bacteria. Two-way ANOVA.

| Source | Nparm | DF | Sum of Squares | F Ratio | Prob > F |
| --- | --- | --- | --- | --- | --- |
| CMD only | 4 | 4 | 5467,051 | 173,8306 | <,0001 |
| CMD+consortia | 1 | 1 | 15737,281 | 2001,533 | <,0001 |
| CMD only*CMD+consortia | 4 | 4 | 4954,854 | 157,5447 | <,0001 |
|  |  |  |  |  |  |
|  |  |  |  |  |  |
| Source | Nparm | DF | Sum of Squares | F Ratio | Prob > F |
| CMD only | 4 | 4 | 2050,508 | 50,2995 | <,0001 |
| CMD+Enterobacter | 1 | 1 | 14900,213 | 1462,026 | <,0001 |
| CMD only*CMD+Enterobacter | 4 | 4 | 2121,204 | 52,0338 | <,0001 |
|  |  |  |  |  |  |
|  |  |  |  |  |  |
| Source | Nparm | DF | Sum of Squares | F Ratio | Prob > F |
| CMD only | 4 | 4 | 1275,031 | 31,861 | <,0001 |
| CMD+Pseudomonas | 1 | 1 | 11349,08 | 1134,383 | <,0001 |
| CMD only*CMD+Pseudomonas | 4 | 4 | 1311,762 | 32,7789 | <,0001 |
|  |  |  |  |  |  |
|  |  |  |  |  |  |
| Source | Nparm | DF | Sum of Squares | F Ratio | Prob > F |
| CMD only | 4 | 4 | 1287,3441 | 160,5166 | <,0001 |
| CMD+Bacillus | 1 | 1 | 5319,8648 | 2653,297 | <,0001 |
| CMD only*CMD+Bacillus | 4 | 4 | 1314,8557 | 163,9469 | <,0001 |

**Supplementary Material Table 2.** Effects of root exudates on macronutrient content in roots. Each nutrient level was analyzed between five treatments using ANOVA. Table shows mean, and standard error (se) calculated from 3 replicates per treatment.

|  | **Nutrient content (mg P root ^-1^)** | | | | | | | | | | | |
| --- | --- | --- | --- | --- | --- | --- | --- | --- | --- | --- | --- | --- |
|  | **N** | **SE (N)** | **P** | **SE (P)** | **K** | **SE (K)** | **S** | **SE (S)** | **Ca** | **SE (Ca)** | **Mg** | **SE (Mg)** |
| **Galactinol** | 3.65 | 0.62 | 1.02 | 0.29 | 10.34 | 0.91 | 1.15 | 0.12 | 2.35 | 0.17 | 2.35 | 0.09 |
| **Threonine** | 2.72 | 0.22 | 0.61 | 0.05 | 10.03 | 1.18 | 1.07 | 0.10 | 2.36 | 0.34 | 2.59 | 0.33 |
| **4-Hydroxybutyric acid** | 2.46 | 0.25 | 0.55 | 0.06 | 9.38 | 0.83 | 1.03 | 0.12 | 2.15 | 0.16 | 2.15 | 0.18 |
| **Mix** | 2.83 | 0.06 | 0.53 | 0.01 | 10.96 | 0.71 | 1.03 | 0.04 | 2.57 | 0.13 | 3.13 | 0.25 |
| **Control** | 2.54 | 0.15 | 0.52 | 0.05 | 9.48 | 1.25 | 0.97 | 0.16 | 2.27 | 0.25 | 2.65 | 0.25 |
| **p-values** | *0.074* | | *0.079* | | *0.251* | | *0.06* | | *0.658* | | *0.844* | |

**Supplementary Material** **Table 3.** Available nutrient content (g/kg) in soils at harvest after periodic application of compounds to soil. Differences were analyzed between treatments for each nutrient found in soil. Different letters denote statistical significance, Tukey HSD test (*p* < 0.05).

|  | **Nutrient content in soil (mg/kg ^-1^ soil)** | | | | | | | | | | | | | | | |
| --- | --- | --- | --- | --- | --- | --- | --- | --- | --- | --- | --- | --- | --- | --- | --- | --- |
|  | **N** | **SE (N)** | **P** | **SE (P)** | **K** | **SE (K)** |  | **S** | **SE (S)** | **Ca** | **SE (Ca)** |  | **Mg** | **SE (Mg)** |  |  |
| **Galactinol** | 0.6 | 0.148 | 7 | 0.297 | 146 | 5.173 | b | 8 | 0.442 | 1530 | 34 | b | 184 | 5.187 | b |  |
| **Threonine** | 0.4 | 0.029 | 7 | 0.440 | 176 | 4.031 | a | 9 | 0.303 | 1731 | 38 | a | 208 | 5.437 | a |  |
| **4-Hydroxybutyric acid** | 0.4 | 0.051 | 7 | 0.172 | 161 | 4.632 | ab | 10 | 0.588 | 1689 | 35 | a | 204 | 3.865 | a |  |
| **Mix** | 0.5 | 0.058 | 6 | 0.169 | 153 | 8.518 | ab | 9 | 0.686 | 1617 | 25 | ab | 196 | 2.676 | ab |  |
| **Control** | 1.0 | 0.312 | 7 | 0.204 | 158 | 5.800 | ab | 10 | 0.477 | 1501 | 41 | b | 182 | 5.183 | b |  |
| ***p-values*** | *0.205* | | *0.185* | | *0.037* | |  | *0.119* | | *<0.001* | |  | *0.002* | |  |  |
